# Analysis of Peristaltic Motion of a Nanofluid with Wall Shear Stress, Microrotation, and Thermal Radiation Effects

**DOI:** 10.1155/2016/4123741

**Published:** 2016-09-05

**Authors:** C. Dhanapal, J. Kamalakkannan, J. Prakash, M. Kothandapani

**Affiliations:** ^1^Department of Science and Humanities, Adhiparasakthi College of Engineering, Kalavai 632506, India; ^2^Bharathiar University, Coimbatore 641046, India; ^3^Department of Mathematics, Priyadarshini Engineering College, Vaniyambadi, Tamil Nadu 635751, India; ^4^Department of Mathematics, Agni College of Technology, Thalambur, Chennai 600130, India; ^5^Department of Mathematics, University College of Engineering Arni, Arni, Tamil Nadu 632326, India

## Abstract

This paper analyzes the peristaltic flow of an incompressible micropolar nanofluid in a tapered asymmetric channel in the presence of thermal radiation and heat sources parameters. The rotation of the nanoparticles is incorporated in the flow model. The equations governing the nanofluid flow are modeled and exact solutions are managed under long wavelength and flow Reynolds number and long wavelength approximations. Explicit expressions of axial velocity, stream function, microrotation, nanoparticle temperature, and concentration have been derived. The phenomena of shear stress and trapping have also been discussed. Finally, the influences of various parameters of interest on flow variables have been discussed numerically and explained graphically. Besides, the results obtained in this paper will be helpful to those who are working on the development of various realms like fluid mechanics, the rotation, Brownian motion, thermophoresis, coupling number, micropolar parameter, and the nondimensional geometry parameters.

## 1. Introduction

Peristaltic pumping is one of the keystones for the development of science and engineering research in modern years. Peristalsis also plays an indispensable role in transporting physiological fluids inside living bodies, and many biomechanical and engineering devices have been designed on the basis of the principle of peristaltic pumping to transport fluids without internal moving parts. The problem of the mechanism of peristaltic transport has attracted the attention of many investigators since the first exploration of Latham [1]. A number of analytical, numerical, and experimental studies on peristaltic motion of different fluids have been described under various conditions with reference to physiological and mechanical environment [2–8].

Micropolar fluids have been a subject of great interest to research workers and a number of research papers have been published on this flow model. Physically, micropolar fluids may represent fluids consisting of rigid, randomly oriented (or spherical) particles suspended in a viscous medium, where the deformation of fluid particles is ignored. This constitutes a substantial generalization of the Navier-Stokes model and opens a new field of potential applications including a large number of complex fluids. Animal bloods and liquid crystals (with dumbbell type molecules) are few examples of micropolar fluids. Local conservation laws of mass, linear, and angular momentum and the energy for polar fluids were received by Grad [9] by using the method of statistical thermodynamics. Eringen [10] proposed the theory of micropolar fluids in which the microscopic effect arises from local structure and fluid elements of micromotion are taken into account. Later, Eringen [11] generalized the micropolar fluids theory to include thermal effects. Using quasi-linearization finite difference technique, an impact of temperature dependent heat sources and frictional heating on the fully developed free convection micropolar fluid flow between two porous parallel plates was analyzed by Agarwal and Dhanapal [12]. Devi and Devanathan [13] premeditated the peristaltic motion of a micropolar fluid in a cylindrical tube with sinusoidal waves of small amplitude travelling down in its flexible wall for the case of low Reynolds number. Srinivasacharya et al. [14] recently examined the peristaltic transport of a micropolar fluid in a circular tube using low Reynolds number and long wavelength assumptions.

Nowadays, there is a continuous focus of the researchers in the flow analysis of nanofluids because of its large number of applications in biomedical and industrial engineering. Choi [15] was the first who initiated this nanofluid technology. A detailed analysis of nanofluids was discussed by Buongiorno [16]. Sheikholeslami et al. [17] studied the natural convection in a concentric annulus between a cold outer square and heated inner circular cylinders in the presence of static radial magnetic field. After initiating a study of nanofluids flow under the effect of peristalsis by Akbar and Nadeem [18], Akbar et al. [19] discussed the slip effects on the peristaltic transport of nanofluid in an asymmetric channel. Recently, Mustafa et al. [20] examined the influence of wall properties on the peristaltic flow of a nanofluid. Mixed convection peristaltic flows of magnetohydrodynamic (MHD) nanofluids were analyzed by Hayat et al. [21]. The effects of wall properties on the peristaltic flow of an incompressible pseudoplastic fluid in a curved channel were investigated by Hina et al. [22]. Hayat et al. [23] studied the peristaltic transport of viscous nanofluid in an asymmetric channel. The channel walls satisfy the convective conditions and also effects of Brownian motion and thermophoresis have also been taken into account. The influence of nanofluid characteristic on peristaltic heat transfer in a two-dimensional axisymmetric channel was discussed analytically by Tripathi and Bég [24]. Moreover, the tremendous applications of nanofluids and the interaction of nanoparticles in peristaltic flows have obtained attentions of many researchers [25, 26].

In the recent years, it is well known by physiologists [27, 28] that the intrauterine fluid flow due to myometrial contractions displays peristalsis and myometrial contractions may occur in both symmetric and asymmetric directions and also noted that blood behaves like as a non-Newtonian fluid in microcirculation [10–12]. Motivated from the above analysis and the importance of peristaltic flows, the purpose of the present paper is to investigate the effects of thermal radiation and heat source/sink on the peristaltic flow of micropolar nanofluids in the tapered asymmetric channel. Therefore, such a consideration of peristaltic transport may be used to evaluate intrauterine fluid flow in a nonpregnant uterus [29]. To the best of the author's knowledge, no attempt is available in the literature which deals with the peristalsis flow of micropolar nanofluid in the tapered asymmetric channel. The present analysis of peristaltic flow is confined to large wavelength and low Reynolds number assumptions. Explicit solutions are developed for axial velocity, axial pressure gradient, stream function, microrotation of the nanofluids, nanofluid temperature, and nanoparticle concentration. The numerical discussion of the pressure rise, shear stresses, and trapping are also obtained and the results are discussed through graphs.

## 2. Mathematical Formulation

**Figure 1:**
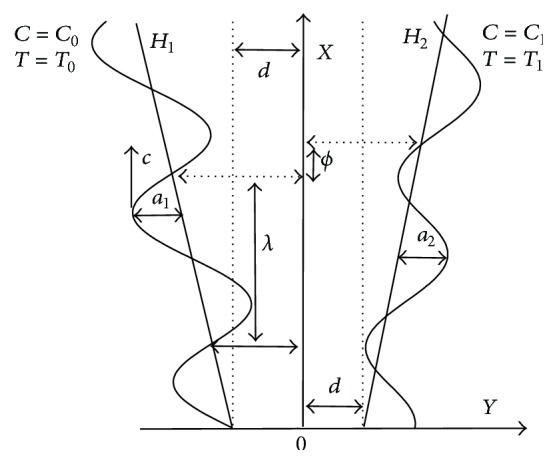
Geometry of the generalized channel (tapered asymmetric channel) with peristaltic wave motion of wall.

Let us consider the motion of peristaltic transport of an incompressible micropolar nanofluid through a tapered channel induced by sinusoidal wave trains propagating with constant speed but with different amplitudes and phases; see Figure 1. The governing equations of motion for the present investigation are [13, 16, 30]

(1) ∂U∂X+∂V∂Y=0,ρf∂U∂t′+U∂U∂X+V∂U∂Y=−∂P∂X+μv+kv∇2U−kv∂W∂Y+1−C0ρfgαT−T0+ρp−ρfgβ′C−C0,ρf∂V∂t′+U∂V∂X+V∂V∂Y=−∂P∂Y+μv+kv∇2V−kv∂W∂X,ρfJ∂W∂t′+U∂W∂X+V∂W∂Y=γv∇2W+kv∂V∂X−∂U∂Y−2kvW,ρc′f∂T∂t′+U∂T∂X+V∂T∂Y=κ∂2T∂X2+∂2T∂Y2−∂qr∂X+∂qr∂Y+Q0T−T0+ρc′pDB∂C∂X∂T∂X+∂C∂Y∂T∂Y+DTρcpTm∂T∂X2+∂T∂Y2,∂C∂t′+U∂C∂X+V∂C∂Y=DB∂2C∂X2+∂2C∂Y2+DTTm∂2T∂X2+∂2T∂Y2,

where *U*, *V* are the components of velocity along *X* and *Y* directions, respectively, *t*′ is the dimensional time, the volumetric volume expansion coefficient is *c*′, *ρ*_*f*_ is the density of the fluid, *ρ*_*p*_ is the density of the particle, *g* is the acceleration due to gravity, *P* is the pressure, *μ*_*v*_, *γ*_*v*_, and *k*_*v*_ are the martial parameters [9–12], *T* is the temperature, *C* is the nanoparticle concentration, *α* is the thermal expansion coefficient, ∂/∂*t*′ represents the material time derivative, *β*′ is the coefficient of expansion with concentration, *W* is the microrotation of the nanofluid, *j* is the microgyration parameter, *T*_*m*_ is the fluid mean temperature, *τ* = (*ρc*′)_*p*_/(*ρc*′)_*f*_ is the ratio of the effective heat capacity of nanoparticle material and heat capacity of the fluid with *ρ* being the density, *κ* is the thermal conductivity of the nanofluids, *D*_*B*_ is the Brownian diffusion coefficient, *D*_*T*_ is the thermophoretic diffusion coefficient, *Q*_0_ is the constant heat addition/absorption, and the radioactive heat flux is *q*_*r*_.

Hence, for the Rosseland approximation for thermal radiation, we have [31, 32]

(2) $$\begin{array}{c} q_{r}=-\frac{4\sigma^{*}}{3k^{*}}\frac{\partial T^{4}}{\partial Y}, \end{array}$$

where *σ* ^*∗*^ and *k* ^*∗*^ are the Stefan-Boltzmann constant and the mean absorption coefficient.

Let *Y* = *H*_1_ and *Y* = *H*_2_ be, respectively, the left and right wall boundaries of the tapered asymmetric channel. Heat and mass transfer along with nanoparticle phenomena have been taken into account. The right wall of the channel is sustained at temperature *T*_1_ and nanoparticle volume fraction *C*_1_ while the left wall has temperature *T*_0_ and nanoparticle volume fraction *C*_0_. The geometry of the wall surface is defined as

(3a) H1X,t′=−d−m′X−a1sin2πλX−ct′+ϕ,

(3b) H2X,t′=d+m′X+a2sin2πλX−ct′,

where *a*_1_ and *a*_2_  are the amplitudes of left and right walls, respectively, *λ* is the wavelength, *m*′ (*m*′ ≪ 1) is the nonuniform parameter, the phase difference *ϕ* varies in the range 0 ≤ *ϕ* ≤ *π*, *ϕ* = 0  corresponds to symmetric channel with waves out of the phase, and further *a*_1_, *a*_2_, *d*, and *ϕ* satisfy the condition for the divergent channel at the inlet of flow

(4) $$\begin{array}{c} a_{1}^{2}+a_{2}^{2}+2a_{1}a_{2}\;cos\left(\;\phi \;\right)\leq {\left(\;2d\;\right)}^{2}. \end{array}$$

The nondimensional parameters are as follows:

(5) x=Xλ,y=Yd,t=ct′λ,u=Uc,v=Vcδ,δ=dλ,h1=H1d,h2=H2d,Ω=dWc,j=Jd2m=m′λd,a=a1d,b=a2d,θ=T−T0T1−T0,σ=C−C0C1−C0,Gr=1−C0ρfgαd2T1−T0cμv,β=Q0d2T1−T0νcp,Pr=μcfκ,Nb=τDBC1−C0ν,Nt=τDTC1−C0T0ν,Rn=16σ∗T033k∗μvcf,Br=ρp−ρfgβ′d2C1−C0cμ,Sc=vDB,p=d2Pcλμ,R=cdρfμv.

Using the above nondimensional quantities in (1)–(4), the resulting equations are

(6) Rδ∂∂t+u∂∂x+v∂∂yu=−∂p∂x+11−NN∂Ω∂y+δ2∂2u∂x2+∂2u∂y2+Grθ+Brσ,Rδ3∂∂t+u∂∂x+v∂∂yv=−∂p∂y+δ21−N−N∂w∂x+δ2∂2v∂x2+∂2v∂y2,Rδj1−NN∂∂t+u∂∂x+v∂∂yΩ=−2Ω+δ2∂v∂x−∂u∂y+2−Nn2δ2∂2Ω∂x2+∂2Ω∂y2,Rδ∂θ∂t+u∂θ∂x+δv∂θ∂y=1Prδ2∂2θ∂x2+∂2θ∂y2+Rn∂2θ∂y2+Nbδ2∂σ∂x∂θ∂x+∂σ∂y∂θ∂y+βθ+Ntδ2∂θ∂x2+∂θ∂y2,RδSc∂σ∂t+u∂σ∂x+δv∂σ∂y=δ2∂2σ∂x2+∂2σ∂y2+NtNbδ2∂2θ∂x2+∂2θ∂y2.

in which *N* = *k*_*v*_/(*μ*_*v*_ + *k*_*v*_) is the coupling number (0 ≤ *N* ≤ 1) and *n* ^2^ = *d* ^2^ *k*_*v*_(2*μ*_*v*_ + *k*_*v*_)/(*γ*_*v*_(*μ*_*v*_ + *k*_*v*_)) is the micropolar parameter. We introduce the nondimensional variables such as *p* is dimensionless pressure, *a* and *b* are amplitudes of left and right walls, respectively, *δ* is wave number, *m* is the nonuniform parameter, *R* is the Reynolds number, *ν* is the nanofluid kinematic viscosity, *Ω* is the dimensionless microrotation, *θ* is the dimensionless temperature, *σ* is the dimensionless rescaled nanoparticle volume fraction, Pr is the Prandtl number, Gr is the local temperature Grashof number, Br is the local nanoparticle Grashof number, Sc is the Schmidt number, *N*_*b*_ is the Brownian motion parameter, *N*_*t*_ is the thermophoresis parameter, and *R*_*n*_ is the radiation parameter as follows. The above equations can reduce to the classical Navier-Stokes equation when *k*_*v*_ → 0.

In several previous attempts [19–24], we employ the long wavelength and low Reynolds number approximations and thus (6) that

(7) ∂p∂x=11−N∂2u∂y2+N1−N∂Ω∂y+Grθ+Brσ,

(8) ∂p∂y=0,

(9) −2Ω−∂u∂y+2−Nn2∂2Ω∂y2=0.

(10) 1+RnPrPr∂2θ∂y2+Nb∂σ∂y∂θ∂y+Nt∂θ∂y2+βθ=0,

(11) ∂2σ∂y2+NtNb∂2θ∂y2=0.

The appropriate boundary conditions are

(12a) u=0,Ω=0,θ=0,σ=0at  y=h1=−1−mx−a sin 2πx−t+ϕ.

(12b) u=0,Ω=0,θ=1,σ=1at  y=h2=1+mx+b sin 2πx−t,

which satisfy, at the inlet of channel,

(13) $$\begin{array}{c} a^{2}+b^{2}+2ab\;cos\;\left(\;\phi \;\right)\leq 4. \end{array}$$

## 3. Exact Solution

By integration of (11) with respect to *y* and implementation in (10) and boundary conditions of (12a) and (12b), the nanoparticles temperature field is obtained as

(14) θ=sinhh1θ1coshθ1y−coshh1θ1sinhθ2ysinhh1θ1coshθ1h2−coshh1θ1sinhθ2h2.

Substituting (14) into (11), moreover integrating (11) with respect to *y* and using proper boundary conditions of (12a) and (12b), the nanoparticle concentration field is received as

(15) σ=h−yNb+BNt sinhh2θ2+ANt coshh2θ1+y−h2NtB sinhh1θ2+A coshh1θ1Nbh1−h2−NtNbA coshθ1y+B sinhθ2y.

Equation (7) can be written in the following form:

(16) ∂2u∂y2=∂∂y1−N∂p∂xy−Nϖ−1−NGrθ−1−NBrσ.

Integration of the above equation yields

(17) ∂u∂y=1−N∂p∂xy−NΩ−1−NGrθ1θ2θ2A sinh θ1y+θ1B cosh θ2y−1−NBr2cy+Dy22−NtNbθ1θ2Aθ2 sinh θ1y+Bθ1 cosh θ2y+Gx.

From (9) and (17), one can write

(18) ∂2Ω∂y2−n2Ω=n21−N2−N∂p∂xy+A4 sinhθ1y+A5 coshθ2y+A6y+A7y2+m2Gx2−N.

The general solution of the above equation can be written as

(19) Ω=E coshny+F sinhny−1−N2−N∂p∂xy+A4 sinhθ1yθ12−n2+A5 coshθ2yθ22−n2−A6yn2−A7n2y2+2n4−Gx2−N.

Making the above equation into (17), one obtains

(20) u=∂p∂xy22−N−A8N sinhnyn−A11N coshnyn−A9N sinhnyn−A12N coshnyn+coshθ1y1−NBrNtANbθ12−1−NGrAθ12−NA4θ1θ12−n2+NA73n2−1−NBrD6y3+sinhθ2y1−NBrNtBNbθ22−1−NGrBθ22−NA5θ2θ22−n2+2A7yNn4+NA6y22n2+Gx2y2−N+A13N coshnyn+A10Nsinhnyn+Hx.

The volume flux through each cross section in the wave frame is given by

(21) $$\begin{array}{c} F=\overset{h_{2}}{\underset{h_{1}}{\int }}u\;dy. \end{array}$$

Using (21), we find that

(22) ∂p∂x=F−A21θ1sinh⁡θ1h2−sinh⁡θ1h1−A22θ2coshθ2h2−coshθ2h1−A32h23−h133−A29nsinhnh2−sinhnh1−A30ncoshnh2−coshnh1−A24h24−h144−A31h22−h124−A25h2−h1·A20h22−h122−N+h23−h136−3N+A27ncoshnh2−coshnh1+A28nsinhnh2−coshnh1+A26h2−h1−1.

The corresponding stream function from (20) is

(23) ψ=∂p∂xy22−N−A8Ncoshny−1n2−A11N sinhnyn2−A9N coshnyn2−A12N sinhnyn2+sinh⁡ θ1yθ1·1−NBrNtANbθ12−1−NGrAθ12−NA4θ1θ12−n2+NA712n2−1−NBrD24y4.

The constant values appeared are listed out in Appendix.

The nondimensional expression for the pressure rise per wavelength Δ*p*_*λ*_ is

(24) $$\begin{array}{c} \Delta P_{\lambda }={\int_{0}^{1}\int_{0}^{1}\left(\frac{\partial p}{\partial x}\right)}_{y=0}dx\;dt. \end{array}$$

Interestingly, we note that the stress tensor in micropolar fluid is not symmetric in behavior. For that reason, the dimensionless form of the shear stress implicated in the present problem under consideration is given by [30]

(25) τxy=∂u∂y−N1−NΩ,

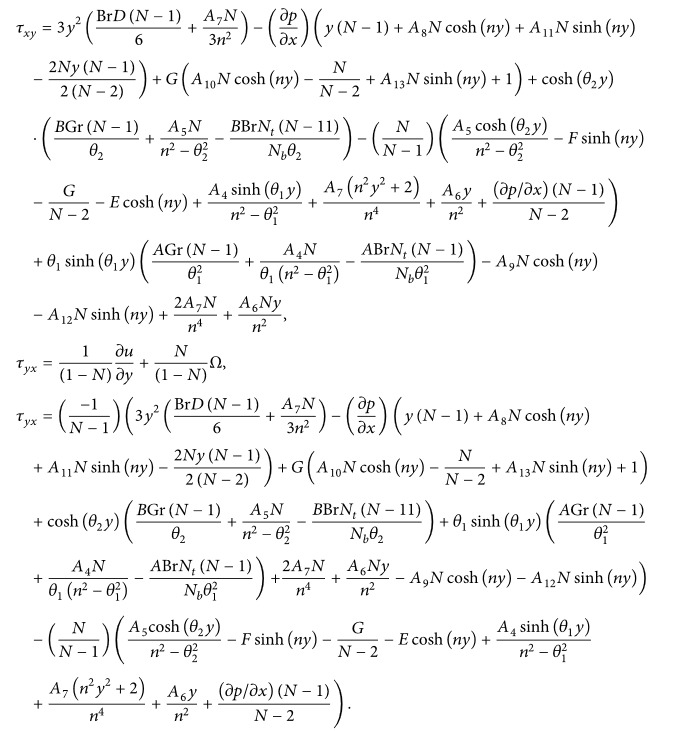

(26)
The numerical computation for the shear stress *τ*_*xy*_ is obtained at left wall of the channel and whose graphical representation is presented in the next section.

## 4. Numerical Results and Discussion

In view of the fact that the constant value of rate of volume flow *F* gives the pressure rise (Δ*P*_*λ*_) always negative and hence pumping action cannot be perceived. To discuss the found results quantitatively, we assume the form of the instantaneous volume rate of the flow *F*(*x*, *t*) [30, 33–37],

(27) $$\begin{array}{c} F\left(\;x,t\;\right)=\Theta +a\;sin\;2\pi \left(\;x-t\;\right)+b\;sin\left[\;2\pi \left(\;x-t\;\right)+\phi \;\right] \end{array}$$

in which $F=\bar{Q}/cd$ , $\Theta =\bar{q}/cd$ , *F* = ∫_*h*_1__^*h*_2_^ *u* *dy* = *ψ*(*h*_2_) − *ψ*(*h*_1_), and Θ is the time-average of flow over one period of wave.

**Figure 2:**
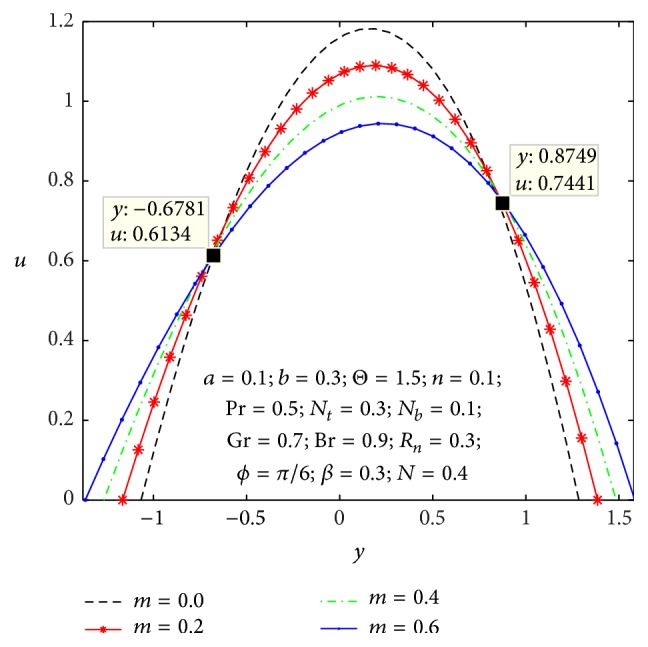
Variation of *m* on the velocity *u* with respect to *y*.

**Figure 3 fig3:**
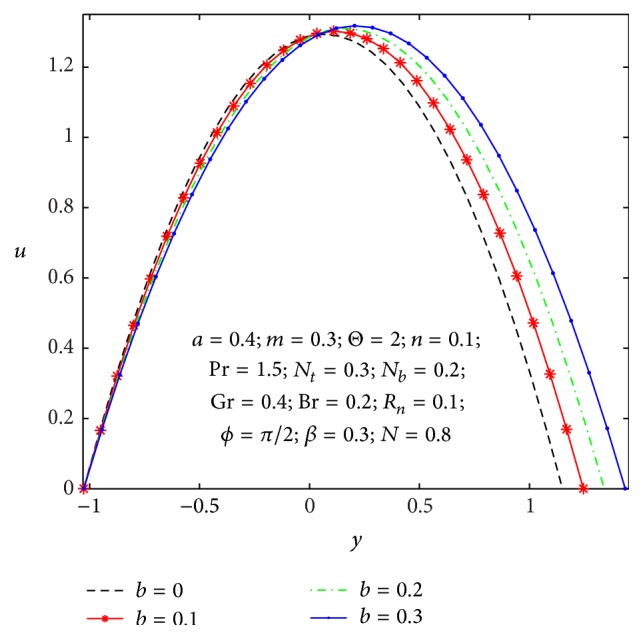
Variation of *b* on the velocity *u* with respect to *y*.

**Figure 4 fig4:**
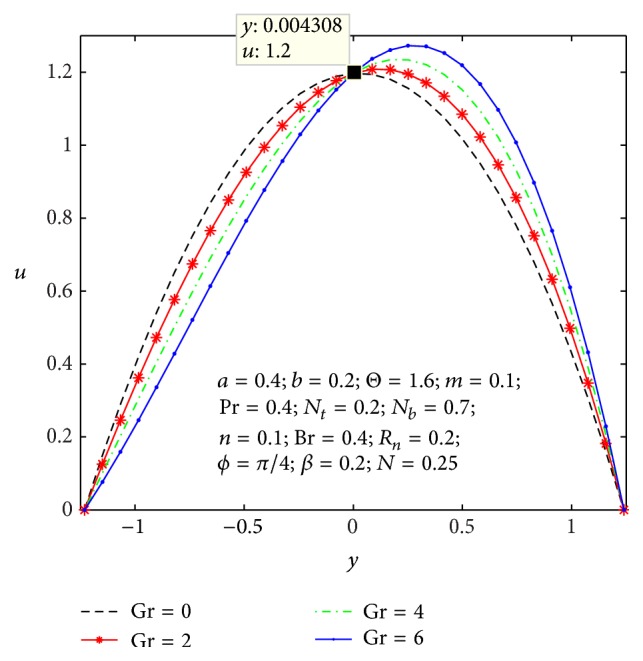
Variation of Gr on the velocity *u* with respect to *y*.

**Figure 5 fig5:**
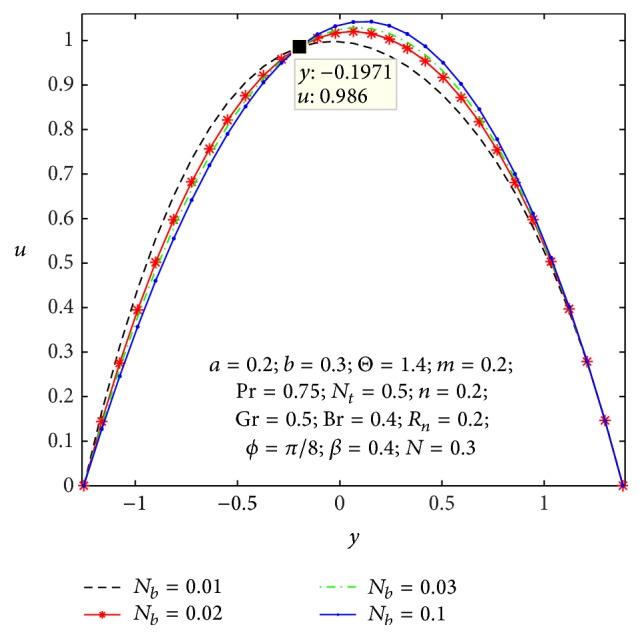
Variation of *N*_*b*_ on the velocity *u* with respect to *y*.

**Figure 6 fig6:**
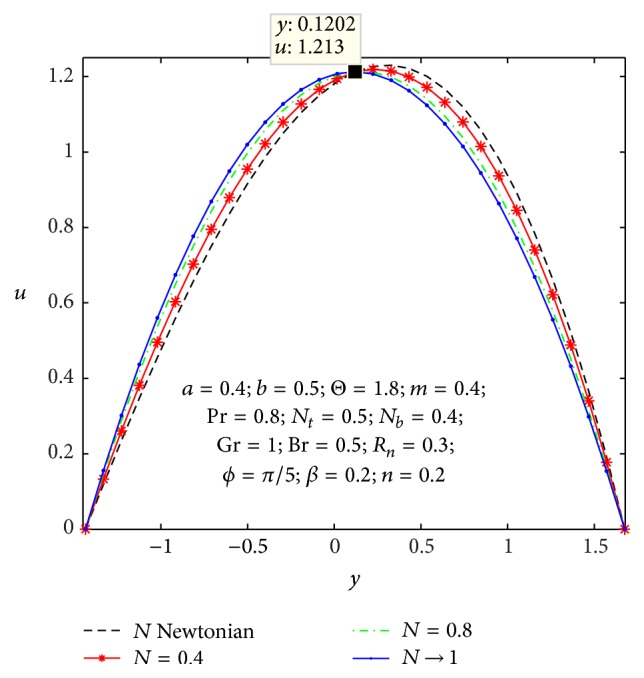
Variation of *N* on the velocity *u* with respect to *y*.

**Figure 7 fig7:**
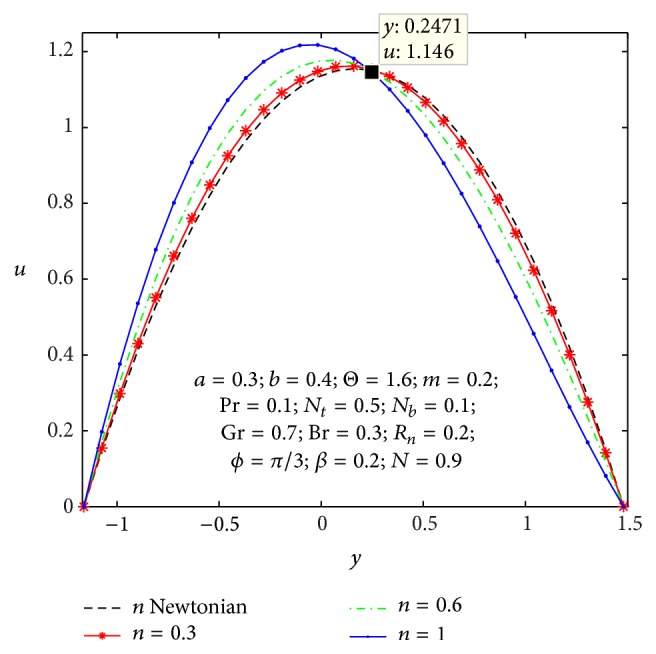
Variation of *n* on the velocity *u* with respect to *y*.

**Figure 8 fig8:**
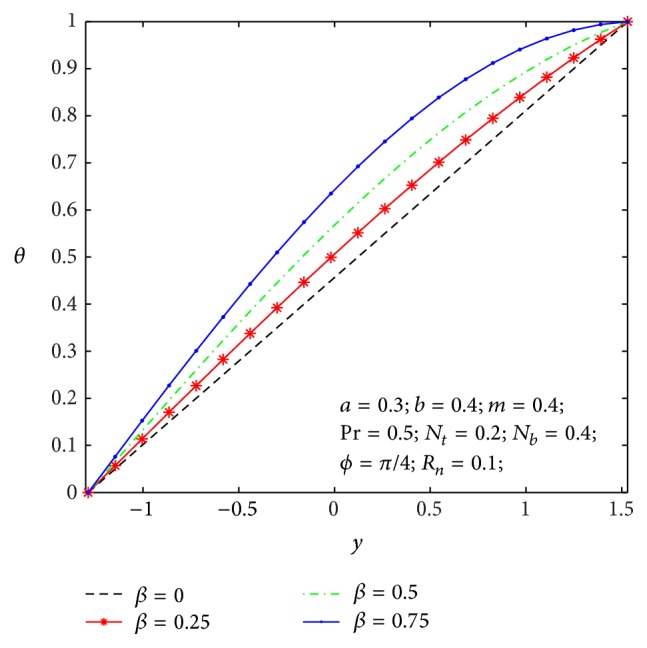
Temperature profile *θ*(*y*) for *β*.

**Figure 9 fig9:**
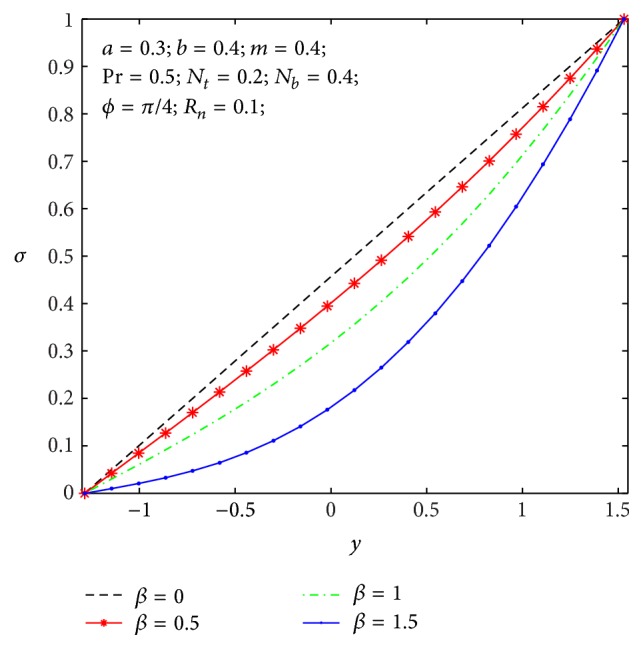
Nanoparticle volume fraction *σ*(*y*) for *β*.

**Figure 10 fig10:**
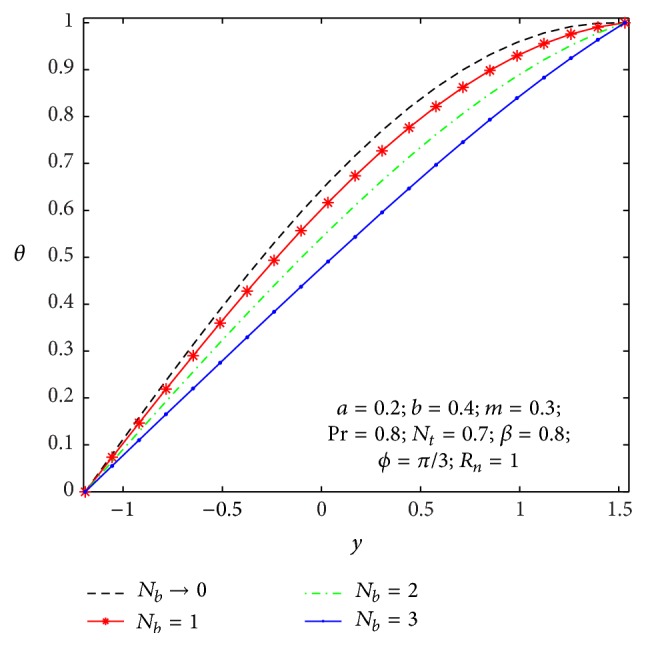
Temperature profile *θ*(*y*) for *N*_*b*_.

**Figure 11 fig11:**
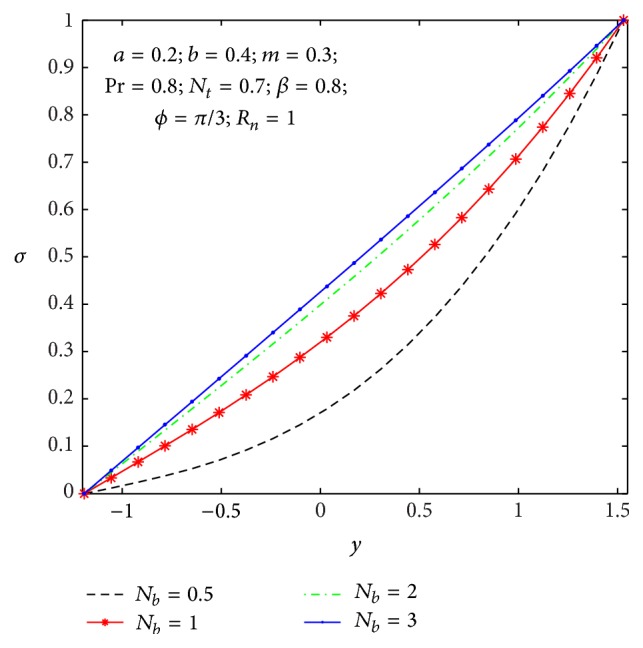
Nanoparticle volume fraction *σ*(*y*) for *N*_*b*_.

**Figure 12 fig12:**
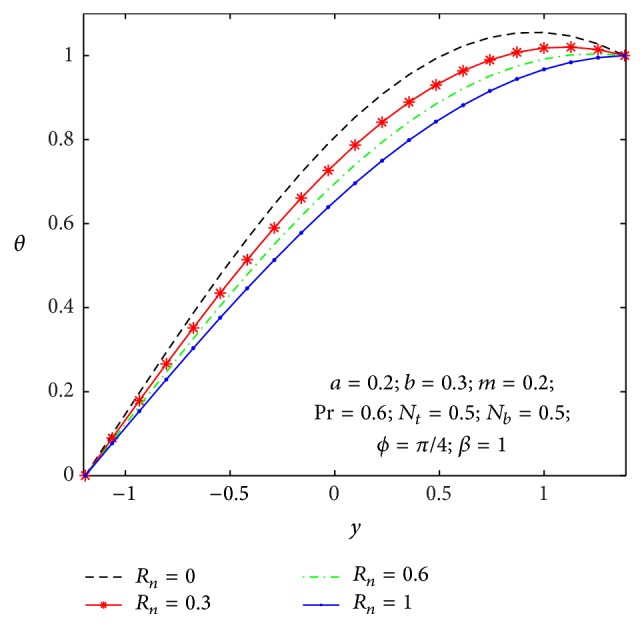
Temperature profile *θ*(*y*) for *R*_*n*_.

**Figure 13 fig13:**
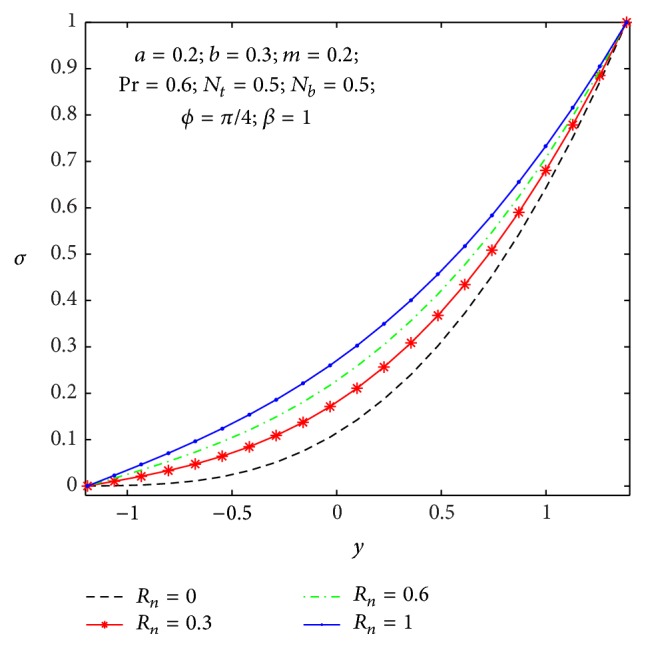
Nanoparticle volume fraction *σ*(*y*) for *R*_*n*_.

**Figure 14 fig14:**
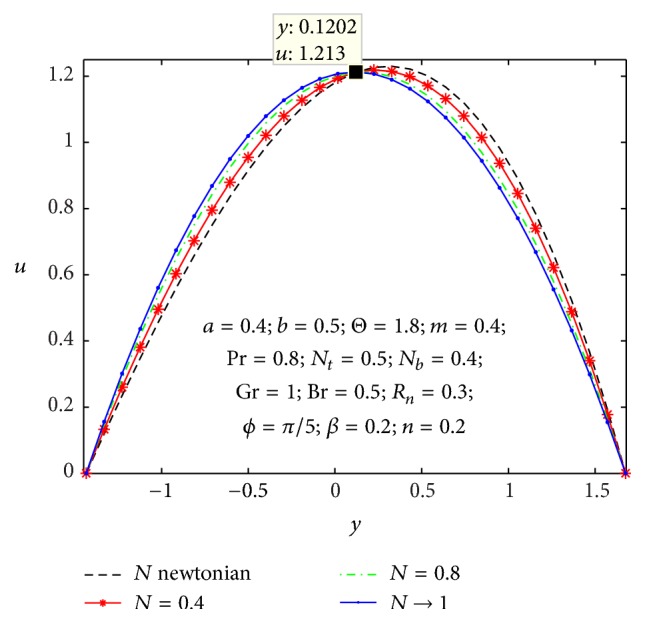
Microrotation velocity profile *Ω*(*y*) for different values of *N*.

**Figure 15 fig15:**
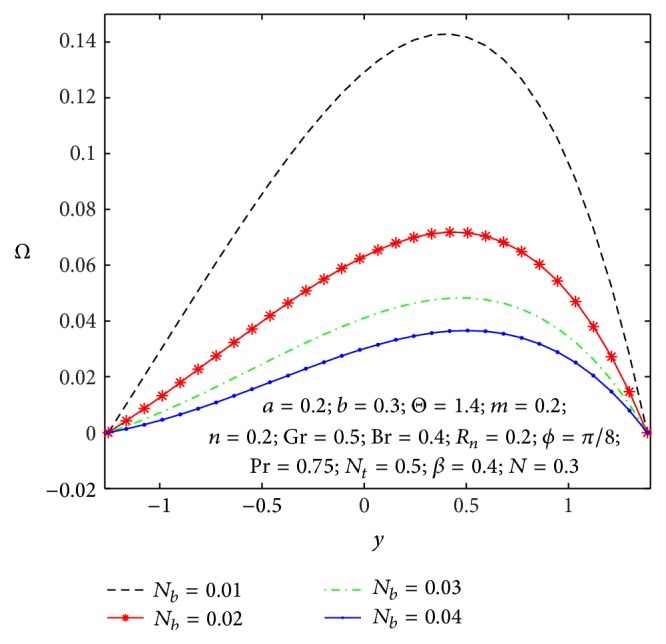
Microrotation velocity profile *Ω*(*y*) for different values of *N*_*b*_.

**Figure 16 fig16:**
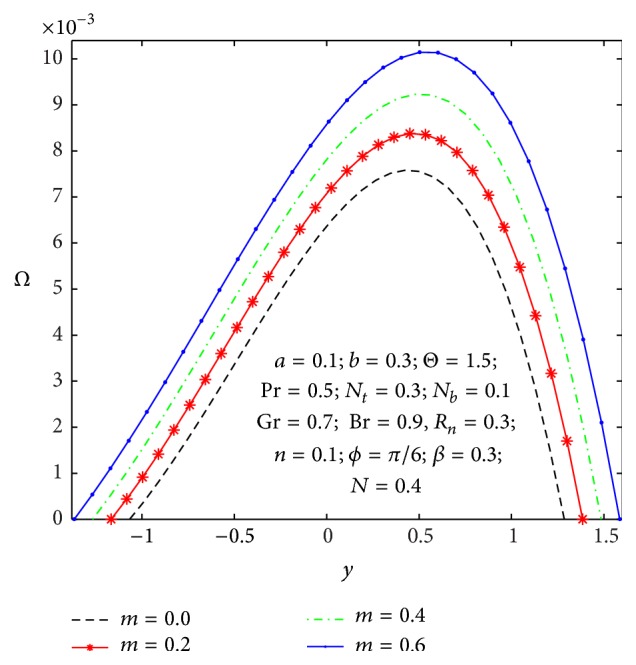
Microrotation velocity profile *Ω*(*y*) for different values of *m*.

**Figure 17 fig17:**
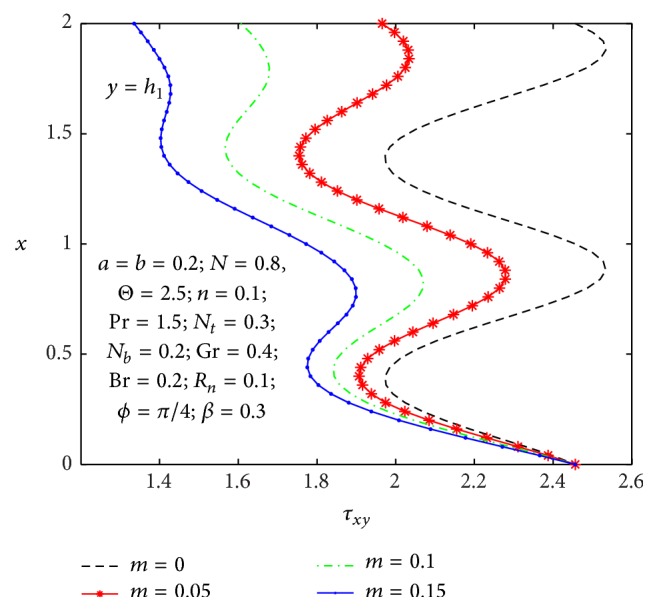
Shear stress profile *τ*_*xy*_(*x*) for different values of *m*.

**Figure 18 fig18:**
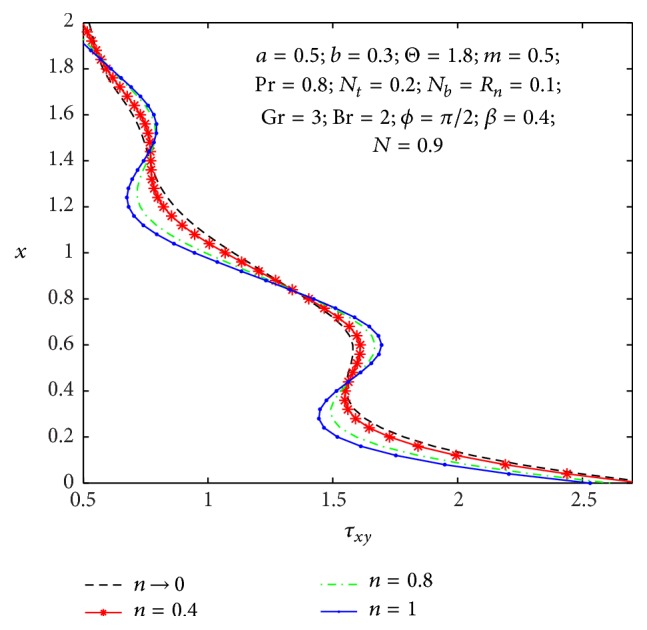
Shear stress profile *τ*_*xy*_(*x*) for different values of *n*.

**Figure 19 fig19:**
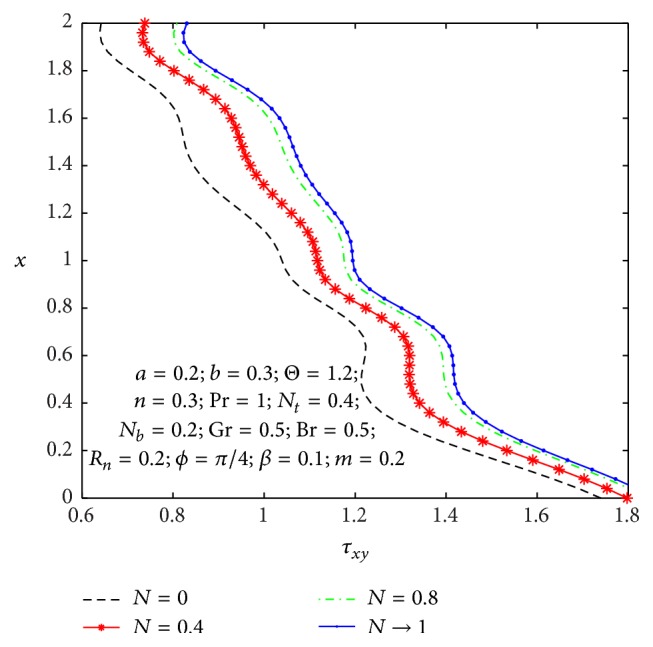
Shear stress profile *τ*_*xy*_(*x*) for different values of *N*.

**Figure 20:**
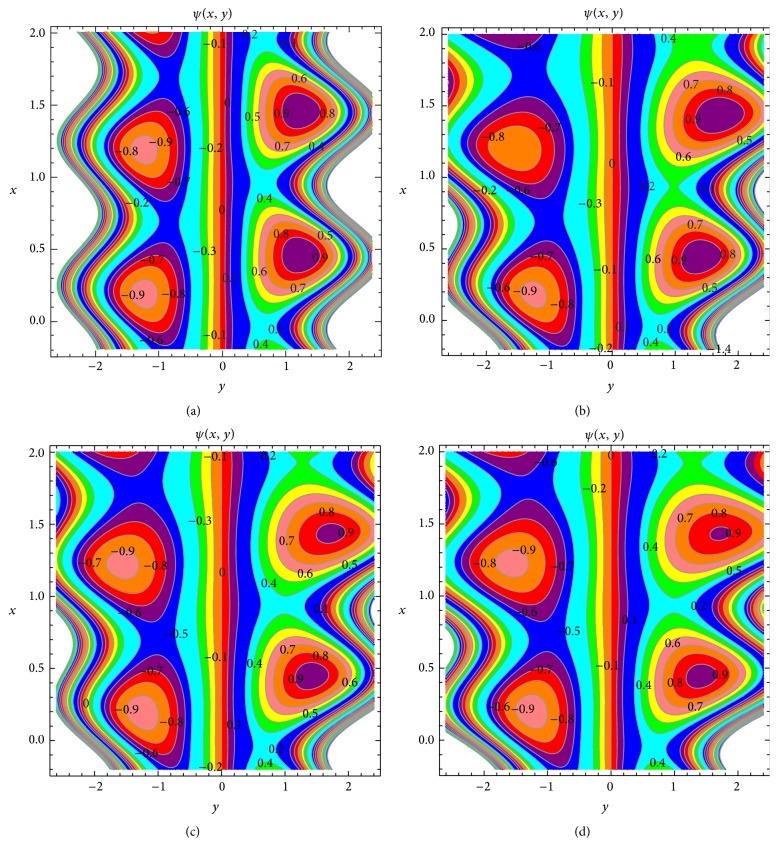
Streamlines for *m* = 0, *N* = 0.01, *n* = 0.1 (a), *m* = 0.3, *N* = 0.01, *n* = 0.1 (b), *m* = 0.3, *N* → 1, *n* = 0.1 (c), and *m* = 0.3, *N* → 1, *n* = 0.9 (d). The other parameters chosen are *a* = 0.2, *b* = 0.3, *m* = 0.3, Θ = 1.5, *ϕ* = *π*/2, *N*_*t*_ = 0.2, *N*_*b*_ = 0.3, *R*_*n*_ = 0.9, Pr = 0.5, Gr = 0.6,  Br = 0.2, *β* = 0.2, and *t* = 0.2.

In order to get nearby into the given substantial problem, we observe physical characteristics of average rise in pressure, axial velocity, microrotation velocity, shear stress, nanoparticles temperature, and concentration with respect of various values to the parameters appearing in the problem by sketching Figures 2 [Fig fig3] [Fig fig4] [Fig fig5] [Fig fig6] [Fig fig7] [Fig fig8] [Fig fig9] [Fig fig10] [Fig fig11] [Fig fig12] [Fig fig13] [Fig fig14] [Fig fig15] [Fig fig16] [Fig fig17] [Fig fig18] [Fig fig19]–20 with the constant values *x* = 0.5 and *t* = 0.2. It is found that, in the absence of nonuniform parameter, local temperature Grashof number, and local nanoparticle Grashof number, the present analysis reduces to approximate analytical solution of peristaltic flow of a Newtonian fluid in asymmetric channel [32]. Thus, the results introduced may be applied in the real life problems associated with nanoparticles movement in the gastrointestinal tract, intrauterine fluid motion induced by uterine contraction, and flow through small blood vessels and intrapleural membranes.

### 4.1. Flow Characteristics

Figures 2 [Fig fig3] [Fig fig4] [Fig fig5] [Fig fig6]–7 express that the variation of axial velocity *u* with respect to *y* for different values of the nonuniform parameter (*m*), amplitude of right wall (*b*), local temperature Grashof number (Gr), Brownian motion parameter (*N*_*b*_), micropolar parameter (*n*), and coupling number (*N*). The dimensionless axial velocity profiles (*u*) satisfy the boundary conditions and are varied by a smooth curve for different (*m*) values in Figure 2. This nonuniform parameter effect has a tendency to slow down the motion of the fluid which results in decreasing the axial velocity profiles at the core of the channel.  Figure 3 represents the variation of *u* with *y* for various values of the amplitude of right wall (*b*). It is found that an increase of *b* results in increase of the velocity of the fluid near the right part of the channel. The effects of Gr and *N*_*b*_ on the axial velocity pattern are shown in Figures 4 and 5. When Gr and *N*_*b*_ values are larger and *u* curves are larger too at the central part of the channel, so the velocity profiles become larger with right wall. This is due to the fact that buoyancy force gives rise to fluid flow. This force has a tendency to accelerate the motion of the fluid which results in increasing the axial velocity profiles at right wall; otherwise it gets decreased. The effect of coupling number *N* on axial velocity is depicted in Figure 6. It is observed that the flow reversal near the left wall of the channel increases with the increase of coupling number, while the reversal trend occurs in the vicinity of the right channel wall. But similar significant change is found for the variation of micropolar parameter *n* in Figure 7.

### 4.2. Heat Transfer and Nanoparticle Mass Transfer Distributions

The dimensionless nanoparticle temperature and concentration profiles (*θ*, *σ*) satisfy the boundary conditions and are varied by a smooth curve for different *β*, *N*_*b*_, and *R*_*n*_ values. In the absence of amplitude of left wall *a* = 0, heat source/sink *β* = 0, Prandtl number *P*_*r*_ = 1, thermal radiation parameter *R*_*n*_ = 0, and nonuniform parameter *m* = 0 our nanoparticle temperature and concentration distributions results are in close agreement with earlier works of Tripathi and Bég [24]. The effects of heat source/sink parameter (*β*) on the nanoparticle temperature and concentration distributions are displayed in Figures 8 and 9. That is, increase in the heat source strength amounts to increase in energy supply to the tapered asymmetric channel and opposite behavior is noticed form nanoparticle mass transfer. The effects of Brownian motion parameter on the nanoparticle temperature and concentration distributions are considered in Figures 10 and 11. When *N*_*b*_ is larger and *σ* curve is lower, so the mass transfer effect is higher for a larger *N*_*b*_ and opposite behavior found in heat transfer. In nanofluid system, the size of the nanoparticle generates Brownian motion which affects the heat and mass transfer properties. As the particle size scale approaches to the nanometer scale, the Brownian motion of particles and its diffusion effect on the liquid play a significant role in heat transfer. For prominent values of *N*_*b*_, the Brownian diffusion effect is large compared to the thermal diffusion effect.  Figure 12 shows the variation of thermal radiation parameter over the temperature field. Therefore higher values of thermal radiation parameter imply higher surface heat flux and so, it decreases the temperature within the tapered asymmetric channel, whereas opposite effects are observed in a nanoparticle mass transfer as shown in Figure 13.

### 4.3. Spin Velocity Distribution

The variation of microrotation velocity (*Ω*) with respect to *y* for various values of coupling number (*N*), Brownian motion (*N*_*b*_), and nonuniform parameter (*m*) are shown in Figures 14 [Fig fig15]–16. Parabolic microrotation velocity profile is observed for the present flow problem. The velocity is maximum or minimum near the center of channel. The cause of coupling number on the microrotation velocity is illustrated in Figure 14. It is seen that in the microrotation velocity increases by increasing the coupling number *N* (i.e., microrotation velocity for the micropolar nanofluid is wider than that of the region for Newtonian nanofluid). In the case of *N* = 0, there is no appreciable difference between Newtonian nanofluids and micropolar nanofluids. The microrotation velocity for the Brownian motion parameter (*N*_*b*_) is plotted in Figure 15. It is seen that, with the increase in the Brownian motion parameter, microrotation velocity profile decreases and maximum microrotation velocity occurs at *N*_*b*_ → 0.  Figure 16 depicts the microrotation velocity field for different values of nonuniform parameter. It is also viewed that the microrotation velocity for a divergent channel (*m* > 0) is higher compared to its value for a uniform channel (*m* = 0).

### 4.4. Shear Stress Distribution

It is well known that the stress tensor is not symmetric in micropolar nanofluid. In Figures 17 [Fig fig18]–19, we have plotted the shear stresses *τ*_*xy*_ at the left wall for values of the nonuniform parameter, micropolar parameter, and coupling number. One can observe from Figure 17 that the wall shear stress decreases with an increase in the nonuniform parameter. From Figure 18, we notice that shear stress is in oscillation behavior, which may be due to the creation of contraction and expansion walls. It is indicated that the shear stress decreases with an increase in the micropolar parameter *n*, while it increases as the coupling number *N* increases in Figure 19. Hence, we observed that the shear stress for a Newtonian nanofluid is less than that for a micropolar nanofluid.

### 4.5. Trapping Phenomena

The effects of nonuniform parameter, coupling parameter, and micropolar parameter on the streamlines are shown in Figure 20. Besides, a comparison between uniform channel and the nonuniform channel is made in Figures 20(a) and 20(b). We note that the size of the trapping bolus increases with increasing nonuniform parameter and symmetry nature is also noticed with respect to uniform symmetric channel. The effects of *N* on trapping are presented in Figures 20(b) and 20(c). This figure reveals that the size of lower bolus decreases with an increase in *N*. From Figures 20(c) and 20(d), it is clear that the trapped bolus increases in size as *n* increases.

## 5. Concluding Remarks

A mathematical model is presented to study wall induced flow of a micropolar nanofluid in the most generalized (tapered asymmetric) channel with the presence of heat source and thermal radiation parameters. The flow model in the rotation of nanoparticles was included. Long wavelength and low Reynolds number assumptions are used in the mathematical modeling. In this investigation, special emphasis has been paid to study the flow features, the axial velocity, the nanoparticles temperature and concentration, the shear stress, and the trapping phenomena. The study leads to the following conclusions:

(i) The axial velocity of fluid decreases at the core part of channel when *m* is increased as anticipated.
(ii) The axial velocity increases near the left wall of channel and decreases near the right wall of the channel with increase of the coupling number and micropolar parameter.
(iii) The nanoparticles mass transfer *σ* has reverse behavior when compared to heat transfer.
(iv) Coupling number and Brownian motion parameters have opposite effects on the microrotation velocity.
(v) The walls shear stress *τ*_*xy*_ decreases with the increase of nonuniform parameter *m* at the left wall but the opposite behavior is identified for coupling number.
(vi) It may be interesting to note that the size of trapped bolus gets increased with increasing of nonuniform parameter.

We ultimately conclude that our theoretical analysis bears the potential to be useful in the field of biomedical and industrial engineering.

## Appendix

The following constants are used in solution section:

(A.1)
θ1=−A2+A22−4A32,θ2=−A2−A22−4A32,A=−sinhθ1h1sinhθ2h2coshθ1h1−sinhθ1h1coshθ1h2,B=coshθ1h1sinhθ2h2coshθ1h1−sinhθ1h1coshθ1h2,C=−Dh1+NtNbA coshθ1h1+NtNbB sinhθ2h1,D=1h2−h1+NtNbh2−h1Acoshθ1h2−coshθ1h1+NtNbh2−h1Bsinhθ2h2−sinhθ2h1,E=A8∂p∂x+A9+GA10,F=A11∂p∂x+A12+GA13,G=A17−A14A16−A19∂p∂x+A18−A15A16−A19,H=−A14+A16A17−A14A16−A19∂p∂x−A15−A16A18−A15A16−A19,A1=Nt+NbNbh2−h1,A2=NbPrA11+RnPr,A3=Prβ1+RnPr,A4=n21−NA2−Nθ1BrNtNb−Gr,A5=n21−NB2−Nθ2BrNtNb−Gr,A6=−1−NBrCn22−N,A7=−1−NBrDn22−N,A8=1−Nh12−Ncoshnh1−1−Nsinhnh1h1 coshnh2−h2 coshnh1sinhnh1−h2coshnh1,A9=−A33coshnh1+coshnh1−coshnh2sinhnh1sinhnh1−h2coshnh1,A10=12−Ncoshnh1−sinhnh1coshnh2−coshnh12−Nsinhnh1−h2coshnh1,A11=1−Nh1cosh⁡nh2−h2cosh⁡nh1sinh⁡nh1−h2,A12=A34 coshnh1−A33 coshnh2sinhnh1−h2,A13=cosh⁡nh2−cosh⁡nh12−Nsinh⁡nh1−h2,A14=h122−N−A8 sinhnh1+A11 coshnh1n,A15=−NA9 sinh nh1+A12 coshnh1n+coshθ1h11−NABrNt−GrNbNbθ12−NA4θ1θ12−n2+sinhθ2h1·1−NBBrNt−GrNbNbθ22−NA5θ2θ22−n2+NA6h122n2+NA73n2−1−NBrD6h13+2NA7h1n4,A16=2h12−N+NA13 coshnh1+A10 sinhnh1n,A17=h222−N−A8 sinhnh2+A11 coshnh2n,A18=−NA9 sinh nh2+A12 coshnh2n+coshθ1h21−NABrNt−GrNbNbθ12−NA4θ1θ12−n2+sinhθ2h2·1−NBBrNt−GrNbNbθ22−NA5θ2θ22−n2+NA6h222n2+NA73n2−1−NBrD6h23+2NA7h2n4,A19=2h22−N+NA13 coshnh2+A10 sinhnh2n,A20=A17−A14A16−A19,A21=1−NBrNtANbθ12−1−NGrAθ12−NA4θ1θ12−n2,A22=1−NBrNtBNbθ22−1−NGrBθ22−NA5θ2θ22−n2,A23=NA73n2−1−NBrD6,A24=A18−A15A16−A19,A25=−A15−A16A24,A26=−A4−A16A20,A27=NA10−A8n,A28=NA13−A11n,A29=NA24A13−A12n,A30=NA24A10−A9n,A31=2A7Nn4+2A242−N,A32=NA62n2,A33=A4 sinhθ1h1θ12−n2+A5 coshθ1h1θ22−n2−A6h1n2−A7n2h12+2n4,A34=A4 sinhθ1h2θ12−n2+A5 coshθ1h2θ22−n2−A6h2n2−A7n2h22+2n4.
